# Novel *mcr-3* variant, encoding mobile colistin resistance, in an ST131 *Escherichia coli* isolate from bloodstream infection, Denmark, 2014

**DOI:** 10.2807/1560-7917.ES.2017.22.31.30584

**Published:** 2017-08-03

**Authors:** Louise Roer, Frank Hansen, Marc Stegger, Ute Wolff Sönksen, Henrik Hasman, Anette M Hammerum

**Affiliations:** 1 Department of Bacteria, Parasites and Fungi, Statens Serum Institut, Copenhagen, Denmark

**Keywords:** antimicrobial resistance, bacterial infections, Escherichia coli

## Abstract

A novel variant of the plasmid-borne colistin resistance gene *mcr-3* was detected on an IncHI2 plasmid in an ST131 CTX-M-55-producing *Escherichia coli* isolate from a Danish patient with bloodstream infection in 2014. The discovery of novel plasmid-borne genes conferring resistance to colistin is of special interest since colistin has reemerged as an important drug in the treatment of infections with multidrug-resistant Gram-negative bacteria.

Very recently, in June 2017, Yin *et al.* reported a new transferable plasmid-borne colistin resistance gene, *mcr-3*, detected on an IncHI2-type plasmid in an *Escherichia coli* isolate from pig faeces in China [1]. The *mcr-3* gene showed 45% and 47% nucleotide sequence similarity to *mcr-1* and *mcr-2*, respectively [1]. Yin *et al.* also compared the *mcr-3* sequence to data from GenBank and found 100% nucleotide similarity to *mcr-3* sequences from a porcine *E. coli* in Malaysia, a human *Klebsiella pneumoniae* isolate in Thailand and a human *Salmonella* Typhimurium in the United States. Furthermore, 99.94% nucleotide similarity was seen in two human *K. pneumoniae* isolates from Thailand [1].

Here we report an *mcr-3* variant from an extended-spectrum beta-lactamase-producing (ESBL) *E. coli* isolated from a bloodstream infection in 2014 in Denmark.

## *mcr-3* in ESBL/AmpC-producing *Escherichia coli* isolates from human bloodstream infections and clinical carbapenemase-producing organisms

Since 2014, ESBL/AmpC-producing *E. coli* isolates from bloodstream infections and all clinical carbapenemase-producing organisms (CPOs) from patients in Denmark have on a voluntary basis been referred to at Statens Serum Institut for whole genome sequencing (WGS) as part of the national surveillance programme DANMAP (www.DANMAP.org).

The 872 ESBL/AmpC-producing *E. coli* isolates from human bloodstream infections collected in the years 2014 to 2016, as well as the 317 human CPOs collected from January 2014 until May 2017 were investigated in silico for the presence of *mcr-3* using MyDbFinder (https://cge.cbs.dtu.dk/services/MyDbFinder/). None of the CPOs were positive for *mcr-3*. 

An *mcr-3*-variant was detected in one ST131 *E. coli* isolate (isolate id SNTR36B6, short read archive (SRA) ID ERR1971735). The isolate was obtained from a male patient admitted to hospital under the clinical diagnosis of pyelonephritis. An ESBL-producing *E. coli* with the same resistance patterns as SNTR36B6 was isolated from catheter-urine (not included in the study). The patient had no former history of hospitalisation and was without known somatic comorbidity. Upon admission, he informed about travel to Thailand two months earlier, where he had stayed locally. Antibiotic treatment with intravenously administered cefuroxime, ciprofloxacin and gentamicin was started at admission. On day 3, the patient had fully recovered, the treatment was changed to monotherapy with perioral ciprofloxacin and the patient was discharged.

In the Sensititre broth microdilution method, the isolate, SNTR36B6, was only susceptible to piperacillin/tazobactam, meropenem, and tigecycline and intermediate resistant to ciprofloxacin according to the European Committee on Antimicrobial Susceptibility Testing (EUCAST) breakpoints [2] (Table 1).

**Table 1:** Minimum inhibitory concentrations and resistance gene profile, ST131 *Escherichia coli* patient isolate carrying an *mcr-3* variant, Denmark, September 2014

Antimicrobial agent	MIC	Interpretation according to EUCAST	Associated resistance gene(s)
Colistin	4	R	*mcr-3*
Piperacillin	> 256	R	*bla*_CTX-M-55_
Piperacillin/tazobactam	2	S	None
Cefotaxime	> 64	R	*bla*_CTX-M-55_
Ceftazidime	16	R	*bla*_CTX-M-55_
Cefepime	> 32	R	*bla*_CTX-M-55_
Aztreonam	32	R	*bla*_CTX-M-55_
Meropenem	≤ 0.03	S	None
Ciprofloxacin	0.5	I	*QnrS1*
Streptomycin	16	^a^	*aadA1, aadA2, strA, strB*
Gentamicin	> 32	R	*aac(3)-Iid, aph(3')-Ic*
Tetracycline	32	R^b^	*tet*(A)
Tigecycline	≤ 0.25	S	None
Trimethoprim	> 32	R	*dfrA12*
Sulfamethoxazole	> 1024	R	*sul3*
Chloramphenicol	64	R	*cmlA1*

EUCAST: European Committee on Antimicrobial Susceptibility Testing; I: intermediate; MIC: minimum inhibitory concentration; R: resistant; S: susceptible.

^a^ No interpretative standards.

^b^ According to the Clinical and Laboratory Standards Institute (CLSI) [16].

The ST131 ESBL-producing *E. coli* isolate, SNTR36B6, had 99.94% nucleotide similarity to the first *mcr-3* gene reported by Yin *et al*. This *mcr-3* variant differed by one amino acid (T488I) from MCR-3 (Table 2). The two *Klebsiella pneumoniae* with 99.94% nucleotide identity to *mcr-3* reported by Yin *et al.* also differed by one amino acid, but at different positions compared with the *mcr-3* (D295E and G373V) in SNTR36B6 [1]. Thus, the *mcr-3* we report here is a novel *mcr-3* variant (Table 2). 

**Table 2:** *mcr-3* and *mcr-3* variants and their deduced MCR-3 and MCR-like proteins in relation to a patient isolate, Denmark, September 2014

Species	Strain	Nucleotide ID^a^	Nucleotide identity with *mcr-3*	Protein ID	Protein identity with MCR-3	Amino acid change	Country	Sample source
*Escherichia coli*	SNTR36B6	ERR1971735	99.94	None	99.82	T488I	Denmark	Human blood
*Escherichia coli*	pWJ1	KY924928	100.00	ASF81896.1	100.00	None	China	Pig faeces
*Escherichia coli*	EC15	NZ_JWKH01000067.1	100.00	WP_039026394.1	100.00	None	Malaysia	Pig vulval swab
*Salmonella enterica serovar* Typhimurium	R9_3269_R1	NZ_NAAS01000133.1	100.00	ORG07507.1	100.00	None	United States	Human stool
*Klebsiella pneumoniae*	PB533	NZ_FLWZ01000042.1	100.00	WP_039026394.1	100.00	None	Thailand	Human pus
*Klebsiella pneumoniae*	PB395	NZ_FLWO01000034.1	99.94	WP_065801616.1	99.82	D295E	Thailand	Human urine
*Klebsiella pneumoniae*	PB517	NZ_FLXA01000011.1	99.94	WP_065804663.1	99.82	G373V	Thailand	Human pus

Modified from Yin et al. [1].

^a^ The ID number for SNTR36B6 refers to the short read archive. The other ID numbers refer to GenBank.

We also investigated our isolates for the presence of *mcr-1* and *mcr-2.* None of the CPOs were positive for either gene. One ESBL-producing *E. coli* strain carried the *mcr-1* gene and has been described earlier [3]. No other *mcr-1*-positive ESBL-producing *E. coli* were detected and none of the isolates were positive for *mcr-2.*

## Plasmid comparison to *mcr-3* plasmid pWJ1

Besides *mcr-3,* we found 12 different resistance genes including *bla*_CTX-M-55_ and *sul3* in SNTR36B6 (Table 2) using ResFinder (https://cge.cbs.dtu.dk/services/REsfinder) [4], and SNTR36B6 was found to carry the *fimH22* allele using FimTyper (https://cge.cbs.dtu.dk/services/FimTyper/) [5].

Using PlasmidFinder (https://cge.cbs.dtu.dk/services/PlasmidFinder/) [6], an IncHI2 replicon was detected in the WGS data from the SNTR36B6 MCR-3-producing *E .coli* isolate. The *mcr-3* gene was initially reported to be located on a 261 kb IncHI2 plasmid, pWJ1, with 18 other known resistance markers [1]. BLAST analysis of the SNTR36B6 sequence against pWJ1, using the GView Server (https://server.gview.ca/), suggested a similar backbone as the pWJ1 plasmid (Figure). The sequence from SNTR36B6 had nine of its 13 resistance genes in common with pWJ1, while nine resistance genes were missing (Figure).

**Figure:**
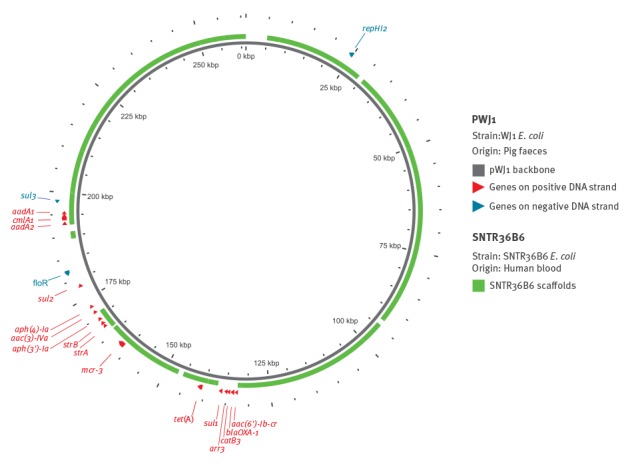
Sequence comparison of *mcr-3* plasmid pWJ1 with *mcr-3*-variant of *Escherichia coli* patient isolate SNTR36B6, Denmark, September 2014

The *mcr-3*-positive *E. coli* SNTR36B6 from human bloodstream infection was compared with the *mcr-3* plasmid pWJ1 using the GView Server. The concentric rings display similarity between the pWJ1 reference plasmid in the inner ring and the SNTR36B3 scaffolds in the outer ring. The red and blue arrows indicate resistance genes of pWJ1, according to the orientation on the DNA strand.

## Discussion

This study is to our knowledge, the first report of *mcr-3* in *E. coli* outside Asia. The fact that an ST131 MCR-3-producing and CTX-M-55 producing *E. coli* isolate was found is of particular concern, since ST131 *E. coli* isolates have spread epidemically during the last decade and the isolate only was susceptible to very few antimicrobial classes such as carbapenems [7,8].

CTX-M-55-producing *E. coli* isolates from humans and animals are commonly reported from Asia [9-11] but are rarely seen in Denmark. However, in 2014 and 2015, CTX-M-55-producing *E. coli* isolates were detected in respectively 3% and 5% of the ESBL/AmpC-producing *E. coli* from bloodstream infections [12]. CTX-M-55 producing *E. coli* isolates were also detected in 2% of the ESBL/AmpC-producing *E. coli* isolates from Danish pigs in 2015 [12]. 

The *sul3* gene was originally detected in a porcine *E. coli* isolate from Switzerland, where 33% of the sulfonamide-resistant porcine *E. coli* isolates carried *sul3* [13]. An investigation of sulfonamide-resistant *E. coli* in Danish pigs, pork and patients from 2002 to 2003 only detected *sul3* in isolates from pigs and pork, but not in human isolates [14]. Between 2014 and 2016, however, the *sul3* gene was detected in 1.5% of the ESBL/AmpC-producing *E. coli* isolates from Danish patients, and was also observed in the SNTR36B6 strain in the present study. 

The *mcr-3* gene was initially reported to be located on an IncHI2-type plasmid named pWJ1. An IncHI2 replicon was also detected in SNTR36B6, and our BLAST analysis suggested that the *mcr-3* variant could be located on a plasmid with a similar backbone belonging to this type, but this will have to be confirmed by further plasmid analysis. However, the lack in SNTR36B6 of several resistance markers which are present on pWJ1 suggests that the plasmid from SNTR36B6 is not completely identical to pWJ1. 

The ST131 *E. coli* isolate carrying the *mcr-3* gene variant in this study, had the *fimH22* allele. Only two isolates with this allele were found among the 122 invasive ST131 ESBL/AmpC-producing *E. coli* isolates in a study from 2017 by Roer et al. [5]. The origin of the ST131 MCR-3-producing and CTX-M-55-producing *E. coli* isolate is unknown, but might be related to travel to Thailand and, based on the presence of the *sul3* resistance gene, it might be of porcine origin.

In conclusion, with the re-emergence of colistin as an important drug in the treatment of infections with multidrug-resistant Gram-negative bacteria [15], the discovery of a plasmid-borne gene conferring resistance to colistin in an *E. coli* of human origin is of special concern. Our findings underline the usefulness of WGS-based surveillance of antimicrobial resistance for detection of new resistance genes by re-analysis of large datasets in silico.
